# Eosinophilic Annular Erythema Responding to Doxycycline

**DOI:** 10.7759/cureus.47478

**Published:** 2023-10-22

**Authors:** Jade N Young, Jina Chung, Nicole Fett

**Affiliations:** 1 School of Medicine, Oregon Health and Science University School of Medicine, Portland, USA; 2 Department of Dermatology, Oregon Health and Science University School of Medicine, Portland, USA

**Keywords:** doxycycline, well's syndrome, eosinophilic dermatoses, eae, eosinophilic annular erythema

## Abstract

Eosinophilic annular erythema (EAE) is a rare skin disease characterized by relapsing and remitting pruritic, annular erythematous plaques and tissue eosinophilia. A 39-year-old male presented with a mildly pruritic, relapsing, and remitting urticarial rash. A biopsy revealed superficial and deep perivascular dermatitis with numerous eosinophils and some neutrophils, with an absence of flame figures. Based on clinical and histopathologic findings, the patient was given a diagnosis of eosinophilic annular erythema. Treatment was initiated with doxycycline 100 mg twice daily. The patient reported substantial improvement at three months and sustained clearance at one year, remaining on doxycycline well tolerated throughout. To our knowledge, no cases of EAE improving with doxycycline have been reported in the literature and, thus, our findings highlight a potential new therapy to consider in a patient with EAE.

## Introduction

Eosinophilic annular erythema (EAE) is a rare skin disease characterized by relapsing and remitting pruritic, annular erythematous plaques and tissue eosinophilia. Approximately 60 cases have been cited in the literature [1]. The pathogenesis of EAE is poorly understood. Current thinking favors the interleukin-5-mediated recruitment of eosinophils in response to an epidermal stimulus, such as insect bites [2]. The histopathology of EAE is classically characterized by a dermal infiltrate of eosinophils, without flame figures [3]. Because of its rarity, there is no standard treatment for EAE. In this article, we describe a case of EAE with a clinical response to doxycycline.

## Case presentation

A 39-year-old male presented to our clinic with a rash comprising mildly pruritic, urticarial plaques that had intermittently come and gone throughout the past nine years. The plaques were previously isolated to his upper back and shoulders but subsequently spread down to his lower back, buttocks, and genitals. During the most recent flare, fixed plaques persisted for one year. During an evaluation eight years prior, he was noted to have a similar rash, fatigue, neutropenia, and thrombocytopenia and underwent a workup for Lyme disease, which was notable for positive anti-borrelia IgM serologies but negative IgG. No history of recent travel or insect bite was described. At that time, he had a biopsy that revealed perivascular and interstitial dermatitis, with numerous eosinophils. He was prescribed intermittent courses of doxycycline ranging from two weeks to three months, during which time the rash would resolve. Several other therapies were trialed, including topical permethrin cream, topical steroids, topical antifungals, famotidine, hydroxyzine, cetirizine, and several food elimination diets, none of which provided a therapeutic response.

**Figure 1:**
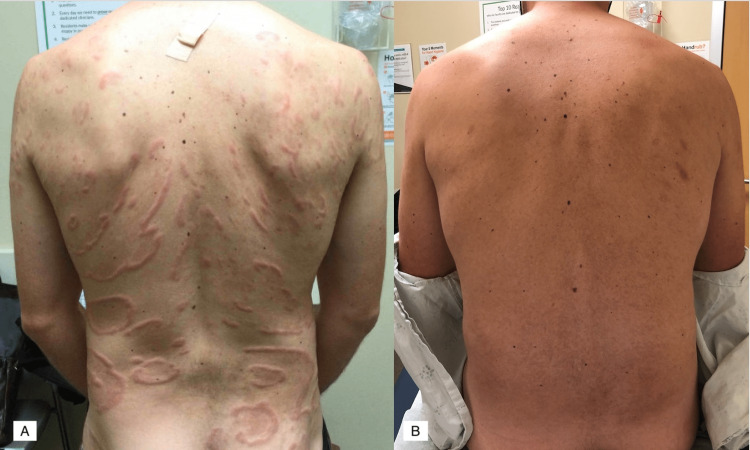
Eosinophilic annular erythema Large concentric, annular, and edematous plaques with some linear and arcuate plaques and papules distributed across the back and right buttock consistent with eosinophilic annular erythema (A), with resolution of rash through a one-year follow-up (B).

**Figure 2:**
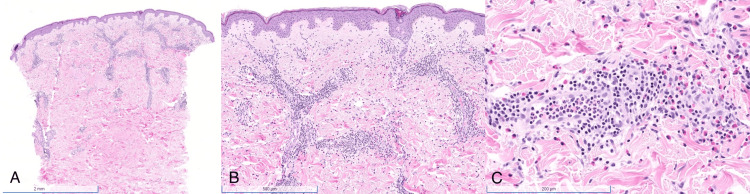
Histopathology (A) There is a punch segment of skin with an unremarkable epidermis and a superficial to mid-dermal inflammatory infiltrate. (B) A closer-up view shows a dense perivascular lymphocytic infiltrate with numerous eosinophils. Flame figures are not seen. (C) The infiltrate surrounding the vessels is composed of lymphocytes, neutrophils, and numerous eosinophils.

On our examination, the rash consisted of multiple large concentric, annular edematous plaques with some linear and arcuate plaques and papules distributed across the back and right buttock, with smaller similar plaques on the trunk, genitals, and upper thigh without excoriations (Figure 1A). Thyroid-stimulating hormone, treponemal antibodies, complete blood count, absolute eosinophil count, and absolute neutrophil count were within normal limits. A biopsy revealed superficial and deep perivascular dermatitis with numerous eosinophils and some neutrophils, with an absence of flame figures (Figure 2). Based on both clinical and histopathologic findings, the patient was given a diagnosis of eosinophilic annular erythema. Treatment was reinitiated with doxycycline 100 mg twice daily, given the previous improvement on this medication. The patient reported substantial improvement three months later and remained clear throughout his one-year follow-up (Figure 1B). Notably, his condition flared during a period of drug cessation while awaiting a refill.

## Discussion

EAE is a rare condition that was first described in 1981 within the pediatric population [4] and has since been characterized in adults [5]. It is debated whether this condition is on a spectrum with eosinophilic cellulitis (EC) or Wells syndrome, as the histopathological findings are distinguished only by the notable absence of flame figures in EAE. The clinical stigmata are distinct, however. Large plaque-like lesions predominate in EC, while figurate, annular lesions present more commonly in EAE [2]. The differential diagnosis for EAE includes EC, erythema annulare centrifugum, erythema gyratum repens, erythema migrans, fixed drug eruption, cutaneous T cell lymphoma, and tumid lupus erythematosus.

Although EAE is typically considered a benign condition with a good prognosis, the relapsing and remitting cycles and associated patient discomfort warrant a therapeutic attempt. Moreover, there is some postulation of systemic implications of EAE, with cases of concomitant autoimmune disease, cancer, and vasculitis being reported in the literature [1,6]. Given there is no standard treatment for this condition, several options have been reported including hydroxychloroquine, systemic corticosteroids, topical steroids, narrow-band UVB, dapsone, and dupilumab [1,7]. To our knowledge, no cases of EAE improving with doxycycline have been reported in the literature. Thus, this report highlights a potential new therapy. While the exact mechanism is unknown, one may posit that the previously described anti-inflammatory properties of doxycycline are responsible for this favorable response [8].

## Conclusions

Although spontaneous resolution cannot be excluded in this patient due to the cyclical nature of this skin disease, repeated flares during periods of doxycycline cessation support a therapeutic benefit. Our findings contribute to the arsenal of available therapies to consider in the approach to patients with EAE.
